# Surgical Options for Pediatric Bilateral Vocal Cord Palsy: State of the Art

**DOI:** 10.3389/fped.2020.538562

**Published:** 2020-12-09

**Authors:** Marilena Trozzi, Duino Meucci, Antonio Salvati, Maria Luisa Tropiano, Sergio Bottero

**Affiliations:** Airway Surgery Unit, Pediatric Surgery Department, Bambino Gesù Children's Hospital, Rome, Italy

**Keywords:** bilateral vocal cord palsy, pediatric, surgical options, voice quality, airway surgery

## Abstract

Management of pediatric bilateral vocal cord palsy (BVCP) is a controversial and challenging topic. It may represent a severe obstructive condition usually associated with respiratory distress, and, in such condition, tracheostomy has been considered the gold standard for a long time. Many surgical options have been described and used to increase the glottic space in BVCP (1), with ongoing research of less invasive techniques. The challenge and current trend in our department and in many major pediatric centers is to avoid tracheotomy through an early treatment. Many techniques introduced in the last decade reduced the number of tracheotomies and increased the decannulation rate. Furthermore, we observed a recent increase in attention to preserve the quality of the voice with new techniques, such as endoscopic arytenoid abduction lateropexy which is in our opinion an important innovation to improve glottic space with satisfactory voice results. We present a review of the literature about the evolution of the treatment options for pediatric BVCP during the years.

## Introduction

Vocal cord paralysis (VCP) is the second most common congenital laryngeal anomaly in pediatric age (10–15%) (2).

Unilateral VCP and bilateral VCP differ in clinical presentation, etiology, and treatment. Bilateral vocal cord paralysis (BVCP) can be characterized by stridor, respiratory distress, suprasternal, and chest retractions, sleep apneas, and failure to thrive, and it represents up to 62% of all pediatric VCPs (3). Instead mild stridor, aspiration, and hoarse and breathy voice are generally suggestive of unilateral vocal cord paralysis (UVCP).

VCP results from laryngeal innervation disorders, posterior glottic stenosis, or cricoarytenoid joint's (CAj's) fixation. All the motility anomalies of the larynx are described with the term “laryngeal immobility.”

An awake laryngeal endoscopy is the essential test for diagnosis of the paralysis, and a complete airway endoscopy under general anesthesia is always recommended to exclude other airway-associated pathologies and to differentiate the paralysis from vocal cord fixation (cricoarytenoid joint ankylosis, posterior glottic stenosis). About 45% of the cases of congenital BVCP have other airway diseases, and the most common are laryngomalacia, subglottic stenosis, and tracheomalacia (4).

In infants and children, VCP has mainly neurological, traumatic (birth trauma), and iatrogenic (post-surgical complications) (4–8) etiology. An MRI is often required to evaluate the central nervous system. In about one-third of the neurological cases, Arnold–Chiari II malformation is present with concomitant hydrocephalus and myelomeningocele. Furthermore, a large number of BVCPs are idiopathic (4, 5) and according to the literature, spontaneous recovery of vocal cord motility is possible within 1 or 2 years over the two-thirds of patients (3, 9, 10).

The treatment's choice has many variables; in particular, urgent intubation may be required in case of severe respiratory distress to ensure a safe airway. Placement of a tracheostomy can be required (11) in order to wait for a spontaneous recovery or the therapeutic choice.

In the last decade, the possibility of an early treatment avoiding tracheotomy has been considered, in particular with the proposal of minimally invasive treatments with preservation of the vocal folds, such as endoscopic arytenoid abduction lateropexy (1) or anterior–posterior cricoid split (12).

The best timing of surgical intervention is not standardized, and it is generally discussed case-by-case. Investigating the etiology of paralysis is crucial to predicting the possibilities of recovery and the timing of treatment. Other important prognostic variables are presence of comorbidities, associated airway diseases, age of the patients, and severity of the clinical condition.

Several surgical procedures to improve the glottic respiratory space have been proposed over the years, both endoscopic and open surgery, but a standard treatment has not been established.

The aim of this paper is a review of the literature on the treatment's options on pediatric bilateral vocal cord paralysis, focusing on the most recent techniques and the most promising and conservative approach currently available in this delicate field.

## Surgical Options

Many surgical options have been described and used to increase the glottic space in BVCP (13), with continuous research of less invasive techniques. In 1946, Woodman (14) published a series of patients treated by arytenoidectomy and suture lateralization of the vocal process performed with the external posterolateral approach. This technique was reported with good results also by Cohen in 1973 (15) and Narcy in 1990 (16).

Other authors described partial and total arytenoidectomy with lateralization of the vocal process through the laryngofissure approach, making a midline incision of the thyroid cartilage (3, 17).

**Figure 1:**
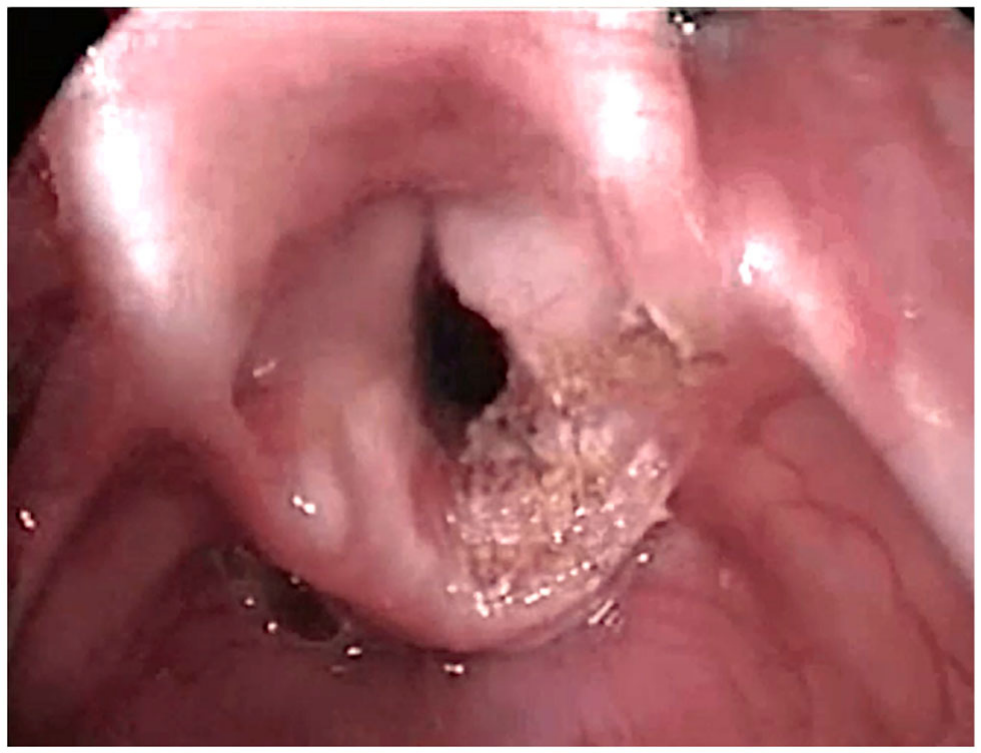
CO_2_ Laser right partial arytenoidectomy.

Thornell was one of the firsts to promote endoscopic procedures; he performed endoscopic arytenoidectomy using electrocautery (18). The development of the CO_2_ laser gave new possibilities, ensuring greater precision, so in 1984 Ossoff et al. (19) described endoscopic CO_2_ laser posterior cordotomy and in 1989 Dennis and Kashim (20) endoscopic laser CO2 arytenoidectomy (Figure 1).

In 1993, Crumley (21) proposed a variation of this procedure: endoscopic laser medial arytenoidectomy, preserving part of the cartilage including the vocal process, to reduce the consequences on the voice function. Bad voice quality is the main adverse effect of the surgery for BVCP, in particular after aritenoidectomy and posterior cordotomy (22, 23).

Most of the literature about the treatment of BVCP is referred to adult patients. One of the few describing a series of pediatric patients compared results of endoscopic vs. open arytenoidectomy [1994, Bower et al. (24)]. The series of 30 patients underwent endoscopic or external arytenoidectomy and lateralization, resulting in a higher decannulation rate for open arytenoidectomy (84%) than the endoscopic arytenoidectomy one (56%).

**Figure 2:**
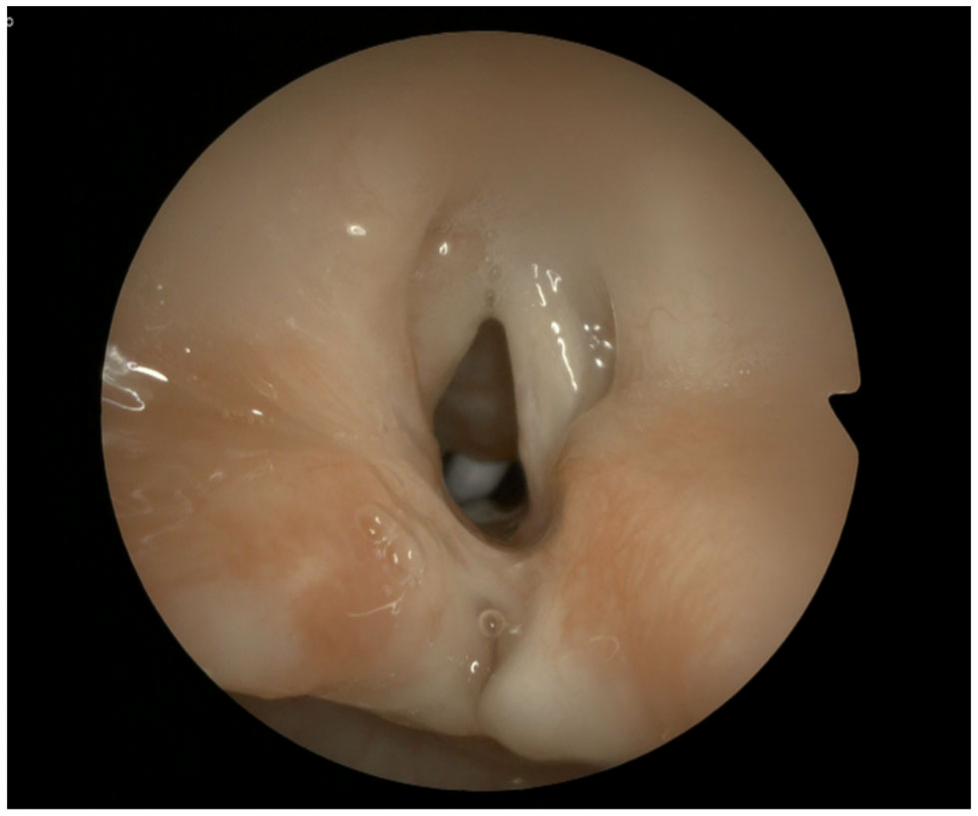
Result after CO_2_ laser left posterior cordotomy.

In 1989, Dennis and Kashima (20) introduced laser partial cordotomy (Figure 2) as a new endoscopic technique, which consisted of a triangular excision of the posterior true vocal fold and false vocal fold to improve the glottic respiratory space. In 2001, Friedman et al. (25) reported a series of pediatric patients all decannulated after posterior cordotomy. A combined endoscopic use of the two methods may be indicated (26).

A surgical revision may be often necessary both post arytenoidectomy than post posterior cordotomy for the presence of scar tissue or granulations, but the decannulation rate is high (27–29).

The use of new technologies has recently increased, with functional improvement and lower risk of thermal damage to the surrounding tissues: in 2015, Googe et al. (30) presented 14 cases of arytenoidectomy by coblator, and in 2018 Basterra et al. (31) described posterior cordotomy in bilateral vocal cord paralysis using monopolar microelectrodes and radiofrequency in 18 patients.

Ozturk et al. (32) compared laser cordotomy and diathermy-assisted cordotomy. Both the techniques gave a sufficient increase in respiratory space, stable over time, confirmed by respiratory functional test results. However, they showed a deterioration in terms of voice quality without significant differences between the two procedures.

**Figure 3:**
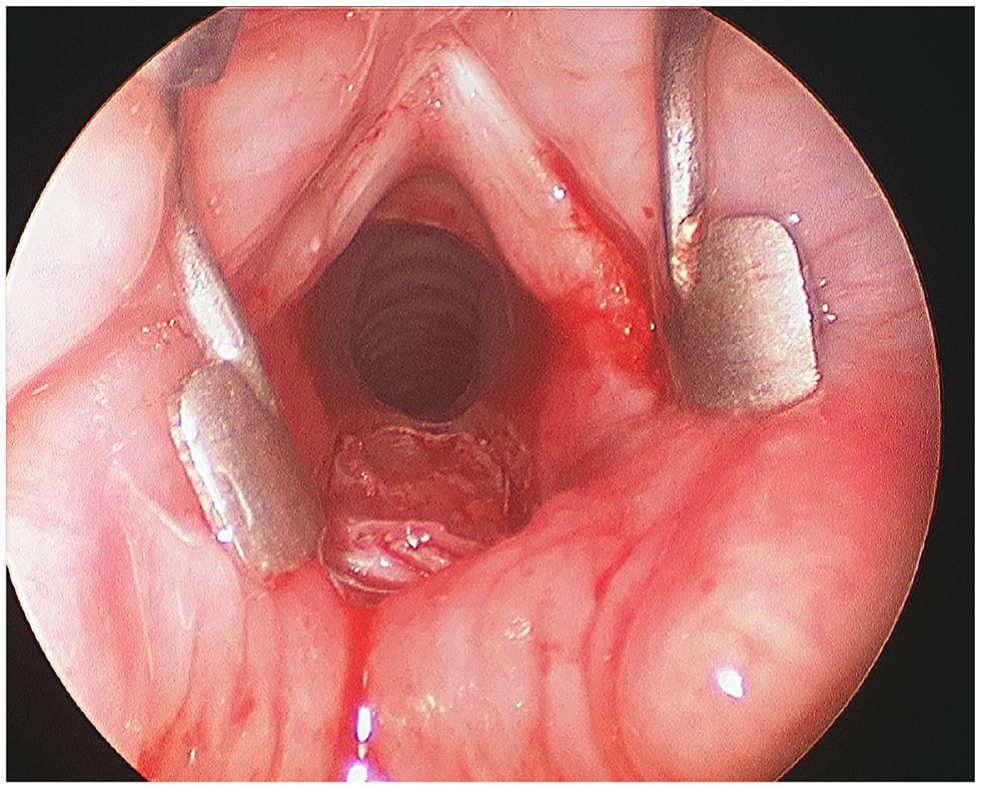
Endoscopic posterior cricoid split with rib grafting.

Another procedure to improve the laryngeal respiratory space is the posterior cricoid split with rib grafting. This surgery, mainly indicated for the treatment of glottic–subglottic stenosis and posterior glottic stenosis (33, 34), consists of a posterior enlargement of the interarytenoid space by splitting the cricoid plate and placing a rib cartilage graft, through laryngofissure (35) or using an endoscopic approach (12) (Figure 3). In 1994, Gray et al. (35) described the treatment of three cases of BVFP using this surgical procedure, with good outcomes in terms of decannulation (each patient was decannulated), and in 2003, Inglis et al. (12) published the results obtained in a series of 10 patients who underwent this surgery through endoscopic technique. For the latter, an excellent laryngeal and subglottic space exposure is crucial to performing midline posterior cricoid incision by CO_2_ laser and splitting and where to put the cartilage rib graft opportunely shaped without appose sutures [no cases of graft dislodgment were described by Inglis in his paper (12)]. Recently, on 2017 again Inglis et al. (36) published a review of their experience about endoscopic posterior cricoid split during the last fifteen years. They described a series of 33 patients (32 had tracheostomy) with different pathologies (subglottic stenosis, posterior glottic stenosis, and bilateral vocal cord immobility) and a rate of decannulation after this surgery of 65.6% (only 28.6% for bilateral vocal fold immobility). This endoscopic procedure has the advantage of preserving the anatomy of the vocal folds and, theoretically, does not cause problems in case of spontaneous recovery of the motility and does not preclude the possibility of other surgeries, but its success was different for the pathologies treated, with worse results in subglottic stenosis combined with vocal cord paralysis. Furthermore, this surgery can be complicated by dysphagia and tracheotomy was considered before or during the endoscopic posterior cricoid split to prevent the risk of respiratory complications in case of dislodgment of the graft (36).

In 2018, Rutter et al. (37) proposed their results with the use of anterior–posterior cricoid split in a series of children in order to avoid tracheotomy. It consists in opening both sides of the cricoid cartilage endoscopically and dilating with an overestimate ETT for about a week. Between 2010 and 2016, they treated 19 patients with this technique and 14 (74%) avoided tracheostomy, demonstrating the effectiveness of the treatment under correct circumstances and so the opportunity to continue with further studies.

In 1922, Rethi (38) described the possibility of glottic enlargement with a lateralization of the vocal cord performed through a laryngofissure. Over the years, this technique has been resumed and perfected with the endoscopic approach as reported by Kirchner et al. in 1979 (39) and Ejnell et al. (40, 41). The laterofixation of the vocal cord is performed, after removal of the thyro-arytenoid muscle, by placing two sutures at the posterior third of the glottis, for each passing the thread above and below the vocal fold and pushing it out through the larynx, up to the skin where they are fixed in a little subcutaneous pouch over the muscle level.

Lichtenberger described the endoscopic vocal cord lateralization (42, 43) performed by using the Lichtenberger needle carrier (44), which more easily allows to pass the thread below and above the vocal fold through the thyroid cartilage, from the endolaryngeal lumen up to the skin. This procedure has been considered reversible from the authors; in case of recovery of vocal cord motility, the suture can be removed and a success rate up to 98% is described in literature (42, 43). Several variations of this technique have been proposed. Mathur et al. (45) tried to simplify the procedure using different instruments to reduce manipulation of the needle and thread inside the larynx and so the risks during the surgery. They performed lateralization of the vocal fold in a series of 10 pediatric patients by a set of spinal needles and a 2–0 stitch-passed extra laryngeal under endoscopic guidance, with a decannulation rate of 100% in the ten patients (45).

**Figure 4:**
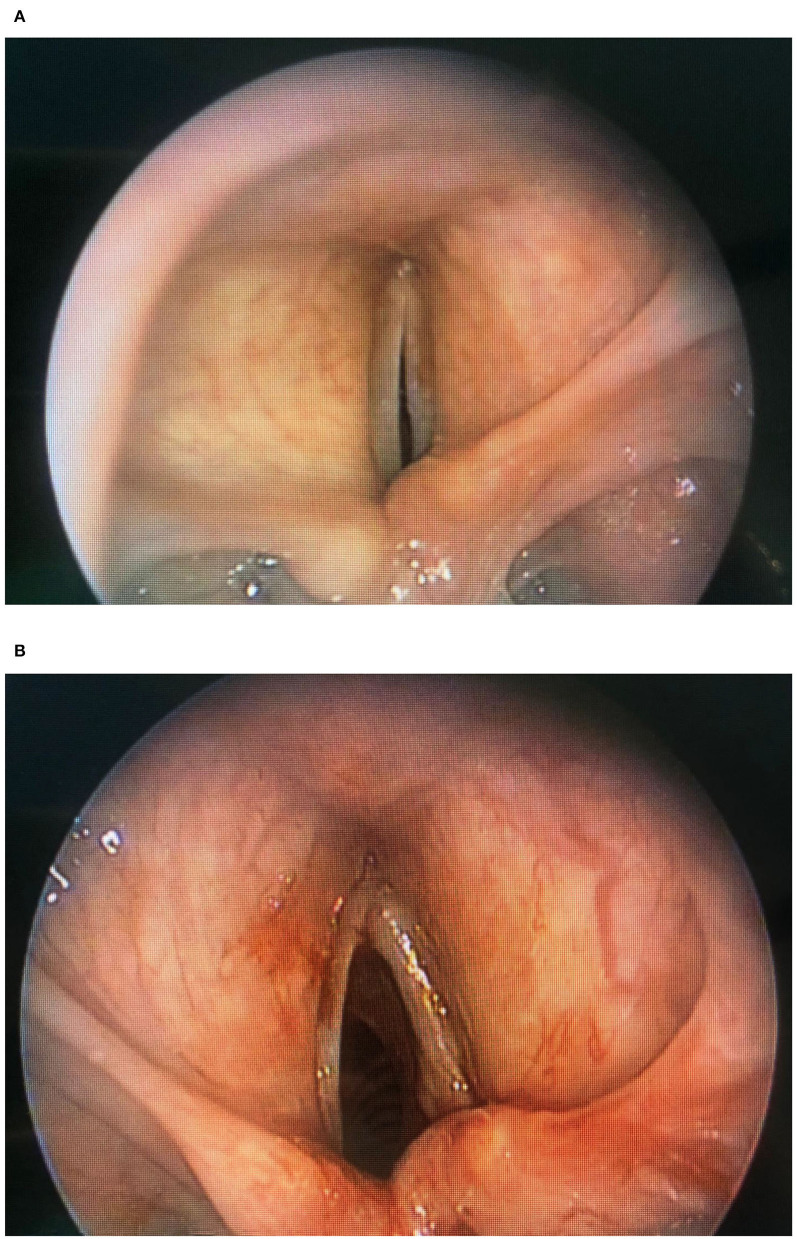
**(A)** Endoscopic arytenoid lateropexy (before surgery). **(B)** Endoscopic arytenoid lateropexy (after surgery).

Woodson et al. (46, 47) described a very interesting alternative to laterofixation: arytenoid abduction. This technique simulates the natural contraction of posterior cricoarytenoid muscle and abduction of the vocal fold (Figures 4A,B). With a transverse skin incision at the midlevel of the thyroid cartilage, the posterior border of the thyroid cartilage is exposed to perform a suture laterofixation of the arytenoid cartilage by a permanent suture placed through the vocal process of the arytenoid and tied.

In 2010, Rovò et al. (48) proposed a new instrument for endoscopic arytenoid lateropexy, a modified endolaryngeal thread guide instrument (ETGI) based on a movable curved blade that guides the thread in and out from the endolaryngeal space up to the skin, with the aim to easily form a loop around the vocal process of the arytenoid cartilage and getting its abduction. They reported the results of the treatment of vocal cord immobility, including 22 adult patients with BVCP, showing how endoscopic arytenoid lateropexy is an effective solution for it (48).

In 2017, Madani et al. (1) described the possibility of using endoscopic arytenoid abduction lateropexy in 4 newborns by a new miniaturized ETGI, with good respiratory outcomes, no dyspnea, or swallowing disorder and satisfactory results about voice quality. Recently, the same Hungarian group (49) evaluated long-term results on the same patients after 3 years from endoscopic arytenoid abduction lateropexy. In terms of breathing, voice, and swallowing, every patient had durable resolution of dyspnea, a normal per os diet, an appropriate growth and development, and a normal voice in 2 cases and slightly impaired voice in 1 case.

Szakács et al. (50) published a comparison of the effects of different endoscopic glottis-widening procedures on cadaver; endoscopic arytenoid abduction lateropexy was the most effective and least destructive on phonation structures, providing with a physiological adduction movement the best phonation closure especially if recovery of adductor function occurs. Furthermore, the authors describe the reversibility of this technique without permanent damage to the vocal folds (49).

None of the surgical techniques above described can restore the normal laryngeal physiological movement of adduction and abduction.

With this purpose, laryngeal reinnervation techniques have been proposed.

In 1974, Miehlike (51) studied on animal model the anastomosis of the distal posterior branch of the laryngeal nerve (innervating the posterior crico-arytenoid muscle) with the main funiculus of the recurrent laryngeal nerve dissected out of the vagus. In 1975, Tucker (52) described a reinnervation procedure in adults consisting in dissecting a branch of the ansa hypoglossi joined to the corresponding area of omohyoid muscle to suture it at the level of the posterior cricoarytenoid muscle. In his series, three of five patients were decannulated with the increased glottic airway in over 80% of patients, but his results have never been reproducible in subsequent studies.

More recently, the use of foreign nerves has been deepened for the laryngeal reinnervation surgery in the treatment of bilateral paralysis. As described by Marina et al. (53) in 2011 and Li et al. (54) in 2013, the phrenic nerve can be used with good results to obtain a branch as a nerve substitute because it has a homogeneous composition of motoneurons involved during inspiration, with improvement of the movements in BVCP up to 93% (54).

Usually in BVFP, obstructive respiratory symptoms are predominant suggesting that adducting movement is partially preserved, so restoring the abduction function is the main purpose of these techniques with the possibility also of transecting the anterior laryngeal nerve branch to reduce the activity of the adductor muscles (53).

Recently, the rerouting of the thyrohyoid nerve for laryngeal reinnervation has been considered (55, 56) to reduce the length of the nerve graft and to find a reasonable alternative when ansa cervicalis is not available as a donor nerve, with good functional results and vocal outcomes (lower GIRBAS scores, higher maximal phonation time, stable, or improved postoperatively voice quality) (56).

Laryngeal reinnervation is a promising technique for BVCP, but the studies are still limited to adults with very limited experience in children (57) and in many cases, they refer to the treatment of unilateral vocal cord paralysis.

Alternative therapies have been investigated in recent years too, such as stem cell implant and gene therapy. Stem cell therapy for tissue regeneration in vocal cord paralysis may be applied by the use of autologous muscle-derived stem cells as Halum et al. (58) proposed in 2007 obtaining in rats the attenuation of muscle atrophy.

In 2016, Dirja et al. (59) tried the use of induced pluripotent stem cells with evidence of good differentiation in muscle cells *in vitro* and 2 weeks of survival in thyroarytenoid muscle of rats. Further papers show that adipose-derived stem cells (60) and olfactory ectomesenchymal stem cells (61) represent an interesting choice for this therapy; in particular, the latter have a high neuroregenerative potential in animal models. Always with the aim of promoting laryngeal nerve regeneration and muscle trophism, the idea of gene therapy has been proposed in several studies. It provides to use genes that encode neurotrophic factors for neuronal regeneration and growth factors for muscle cell proliferation and differentiation (62). These genes are linked to vectors that are injected into the laryngeal nerve and muscles (63, 64).

In 1998, Shiotani et al. studied the effect of insulin-like growth factor I gene in rats' denervated larynges (65), reporting an improvement of the size of muscle fibers and the number of axon terminals in the thyroarytenoid muscle, and in 2007 the effect of an adenoviral vector-encoding glial cell line-derived neurotrophic factor (GDNF) on the nucleus ambiguus of rats with vagus nerve injury (66), showing a lower loss of motoneurons after this treatment.

Both gene therapy and stem cell therapy are still experimental with *in vitro* and animal studies, therefore requiring further research and finding the solution to some problems such as ineffectiveness in preventing synkinesis, the risk of damage by viral vectors, and poor cell culture survival (51, 67).

Currently, functional electrical stimulation is another approach in progress for BVCP treatment. In 2016, Mueller et al. (68, 69) described the results of laryngeal pacing implant in 9 patients with evidence of spirometric parameter improvement, in particular of peak expiratory flow, without negative effects on the voice quality and swallowing. The first who introduced the functional electrical stimulation were Zealear and Dedo in 1977 (70), acting on unilaterally paralyzed cricothyroid muscle of the canine. In the following years, other authors reported the results about the stimulation of paralyzed posterior cricoarytenoid muscles, always in the canine model (71–73). Then in 1996, Zealear et al. (74) described the possibility of laryngeal pacing in humans, presenting in 2003 the results of unilateral laryngeal pacing in 7 patients with BVCP using an external device (75). In 2019, Muller and Pototschnig (76) showed their technique of electrical stimulation of the recurrent laryngeal nerve: one of the pacer electrodes is inserted into the PCA muscle, and the pacer is placed in a subcutaneous pocket over the sternum with another electrode on the lateral chest. Furthermore, they underline the importance of selection of patients who could have benefits from this procedure, as, for example, the presence of aberrant or synkinesis reinnervation is essential to ensuring good clinical performance of laryngeal pacemakers, although it is commonly considered an unfavorable condition for recovery of vocal cord movement (76). This technique has a high potentiality, ensuring a ventilatory improvement without compromising voice and swallowing (67). It could overcome the other surgical approach (76), and it has been described as superior to posterior cordotomy in ventilation and voice outcomes by Li et al. (77). However, it is still a complicated experimental procedure, more expensive than other surgeries considering also that the device must be replaced at least every 10 years (66), and so far there is no pediatric experience.

The comparisons of the different techniques do not show significant differences in literature, as reported by Gupta et al. (78), who found similar results in the decannulation rate between laser cordectomy, arytenoidectomy with fold lateralization, and endoscopic fold lateralization in a series of 61 patients (adults and children).

Partial arytenoidectomy and posterior cordotomy give an immediate respiratory space enlargement as well as vocal cord laterofixation, but the latter is certainly more conservative for laryngeal anatomy.

Laterofixation can be considered for its minimal invasiveness and reversibility (67, 79), ensuring minor impairment of voice. In particular, endoscopic arytenoid abduction lateropexy, characterized by an arytenoid abducted movement that preserves the vocal cordal anatomy and therefore the integrity of the mucosal wave, is a valid approach to obtain good respiratory outcomes preserving a good voice quality. During the last 2 years, at our Airway Surgery Unit, we focused the attention on this technique with excellent results that we are analyzing for an upcoming publication, characterized by significant improvement of the respiratory space and high decannulation rate maintaining a good quality of voice.

After diagnosis of BVCP, time of observation may be indicated especially in patients in whom spontaneous recovery can be expected (79), but the challenge and current trend in our department and in many major pediatric center is to avoid tracheotomy through an early treatment (67, 79). Our opinion, supported by literature (48), is that the endoscopic arytenoid abduction lateropexy is a good approach for an early treatment also in infants considering its characteristics of preservation of the vocal cord anatomy and reversibility in case of spontaneous recovery.

Exactly to evaluate the possibility of spontaneous recovery, laryngeal electromyography could have an important prognostic role identifying the candidates for early intervention, as described in several studies (80–84). Laryngeal electromyography shows the motor unit potentials, through hooked wire electrodes inserted into the thyro-arytenoid muscle and the posterior cricoarytenoid muscle, and it can be used to differentiate between mechanical (i.e., cricoarytenoid joint fixation) and neurological impairment of vocal fold and to localize the neuromuscular lesion.

As reported in a recent paper by Giotakis and Pototschnig (85), the presence of volitional activity in the multiple LEMG findings indicates the possibility of a full late recovery of laryngeal motility, allowing to select the cases for a wait-and-see strategy or a reversible approach, rather than a permanent laryngeal surgery. However, in the literature the opinion about the predictive value of this test is under debate, with data ranging from 13 to 96% (80, 81, 86).

## Summary

Identifying the best surgical approach for BVCP in pediatric patients is not easy, considering also that the experiences reported by many authors concern adult patients with very few and small series in children and that the comparisons of the different techniques do not show significant differences in literature.

Most studies report the results in terms of respiratory improvement and decannulation rate, with less attention to voice quality evaluation or assessing swallowing, even if common side effects of the BVCP surgery are dysphonia, dysphagia, and aspiration, but comparing with previous reviews in pediatrics' BVCP treatments, in addition to the global trend to reduce invasiveness, we observed a recent increasing in attention to preserve the quality of the voice.

The new techniques and therapies described above still require further studies and trials but are promising for the future in order to overcome more destructive surgical techniques and giving a potential dynamic functional recovery of the larynx.

## Author Contributions

MT, DM, AS, MLT, and SB contributed conception and design of the study. MT, DM, and AS wrote the manuscript. All authors contributed to manuscript revision, read and approved the submitted version.

## Conflict of Interest

The authors declare that the research was conducted in the absence of any commercial or financial relationships that could be construed as a potential conflict of interest. The handling editor declared a past co-authorship with one of the authors MT.
